# Co-occurrence of radiological signs of Marchiafava-Bignami disease and alcohol-related cerebellar degeneration

**DOI:** 10.1590/1980-5764-DN-2024-0216

**Published:** 2025-04-07

**Authors:** Alberto Pereira Firmino, Maria Weryca de Souza Belo Silva, Beatriz Barbosa, Enrique Neves, Letícia Ellen Pereira, Thadeu Alexandre Paulino Sousa, Katie Moraes de Almondes, Clécio de Oliveira Godeiro, Rodrigo Alencar e Silva

**Affiliations:** 1 Universidade Federal do Rio Grande do Norte, Hospital Universitário Onofre Lopes, Natal RN, Brazil.; 2 Hospital Universitário Onofre Lopes, Serviço de Neuropsicologia do Envelhecimento, Natal RN, Brazil.; 3 Hospital Monsenhor Walfredo Gurgel, Natal RN, Brazil.; 4 Universidade Federal do Rio Grande do Norte, Departamento de Psicologia, Natal RN, Brazil.; 5 Universidade Federal do Rio Grande do Norte, Programa de Pós-Graduação em Psicobiologia, Natal RN, Brazil.; 6 Universidade Federal do Rio Grande do Norte, Hospital Universitário Onofre Lopes, Departamento de Neurologia, Natal RN, Brazil.

A 53-year-old man with a history of chronic alcohol consumption developed anterograde amnesia, accompanied by visual and auditory hallucinations and behavioral changes. Cognitive evaluation revealed mild impairment in the frontal battery and significant decline in episodic memory (Table 1). On physical examination, the patient presented asymmetric cerebellar ataxia, more pronounced on the left side, along with global areflexia. External eye movements were preserved, and no nystagmus was observed.

**Table 1:** Neuropsychological assessment.

53 years old, 7 years of schooling, retired (bricklayer)				
Tests	Patient's score	Z score	Interpretation	Date
Frontal Assessment Battery	15/18		Mild Significant Impairment	07/04/2024
Apraxia Tests	22/22		Without apraxia Without astereognosis	07/04/2024
Digit Span	Forward Span total:7 span:5	Forward Span total:0.06 span:0.09	No impairment evidenced in attention or working memory.	07/04/2024
	Backward Span total:4 span:3	Backward Span total:-0.25 span:-0.41		
Rey Auditory Verbal Learning Test (RAVLT)	ΣA1A5: 17	ΣA1A5: −2.95	Patient showed clinically significant decline in episodic memory.	07/04/2024
	A7: 4	A7: −1.71		
	Recognition: −1	Recognition: −2.28		
	Learning Over Trials: 17	Learning Over Trials: 0.18		
	Forgetting Speed Index: 1.33	Forgetting Speed Index: 1.63158		
The Stick Design Test	12/12		No evident impairment in visuoconstructive skills.	07/04/2024
Five Digit Test	Counting time: 55 errors: 0	Counting time: −3,87 errors: 0	Inconclusive result. Patient was unable to complete the testing due to difficulties with visual acuity.	07/04/2024
The Geriatric Depression Scale (GDS-15)	9/15		Mild Depressive Symptoms	07/25/2024
Boston Naming Test (BNT) - Reduced Version	14/15	0.52941	No apparent impairment in semantic memory	07/25/2024
F-A-S Phonemic and Semantic Verbal Fluency Test	Semantic: 17	Semantic: 0.30238	Without evidence of impairment in semantic memory nor executive functions	07/25/2024
	Phonemic: 36	Phonemic: 0.48704		
Psychological Battery for Attention Assessment (BPA)			The patient was unable to perform due to severe tremors.	07/25/2024
Benton Visual Retention Test (BVRT)			The patient was unable to perform due to severe action tremors and low visual acuity	07/25/2024

**Figure 1:**
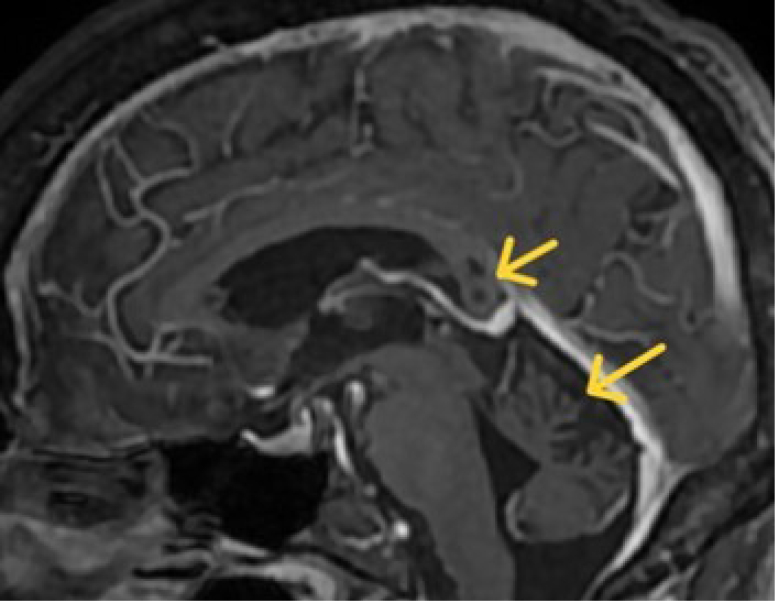
Brain magnetic resonance imaging sagittal T1 sequence, post-contrast, showing hypointensity of the splenium lesion and cerebellar vermis atrophy, no enhancement by the contrast.

**Figure 2:**
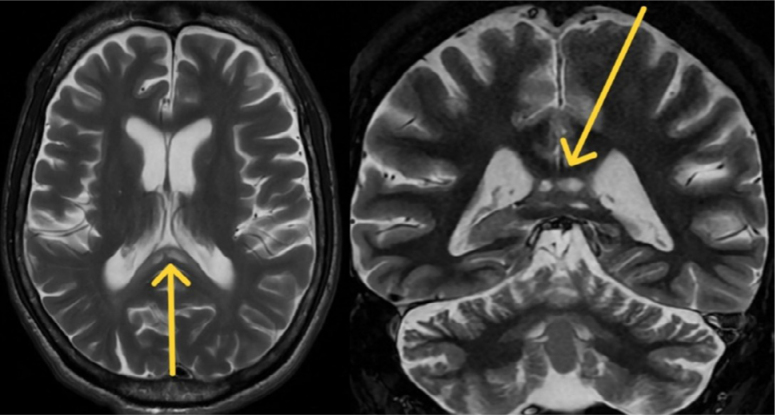
Brain magnetic resonance imaging axial and coronal T2 sequences showing hyperintense lesions, without mass effect, involving the splenium of the corpus callosum.

Brain magnetic resonance imaging (MRI) reveals global atrophy, predominantly affecting the cerebellar vermis, with three lesions exhibiting hypointensity on T1 and hyperintensity on T2 in the splenium of the corpus callosum (Figures 1 and 2). No restriction in water diffusion or enhance with gadolinium was noted.

Marchiafava Bignami Disease (MBD) is a disorder associated with vitamin deficiency due to chronic alcohol abuse or malnutrition. It classically leads to signal changes in the corpus callosum, as seen on MRI, associated with neuropsychiatric symptoms^1^. Despite its rarity, knowledge of its clinical and radiological aspects is crucial for early suspicion and accurate diagnosis, which can help prevent negative outcomes.

Some authors advocate classifying the clinical presentation into at least two distinct syndromes, based on the time of onset, severity of symptoms, as well as neuroimaging findings. In this sense, an acute, more severe form is characterized by faster progression and greater involvement of the level of consciousness, often associated with pyramidal symptoms, seizures, and eventually death. Chronic cases, such as the one described above, typically present with slower clinical onset, gait disturbances, dysarthria, interhemispheric disconnection, less pronounced involvement of awareness, and better prognosis^2,3^.

The typical presentation involves lesions with a cystic appearance or an edema/demyelinating substrate, generally located in the corpus callosum, which may also affect the deep white matter. The number and characteristics of the lesions may change with disease progression. Fewer lesions, restricted diffusion, and gadolinium enhancement typically indicate an acute process^2,4,5^.

Chronic alcohol consumption has long been associated to structural damage to brain tissue, leading to a variety of neuroimaging patterns, with MBD being one of the many possible complications of this deleterious behavior. A classic feature in patients with chronic alcoholism is brain volume loss, particularly in the cerebellum and frontal lobe. In addition, involvement of the pontocerebellar fibers may lead to pontine atrophy. Less commonly, striatal degeneration may be observed in some subjects, and the combination of MBD and Wernicke encephalopathy has also been described. This supports the statement that, as in the case described above, other findings associated with alcohol abuse, such as cortical and cerebellar atrophy, may be present simultaneously^1,6^.

This overlap with other, sometimes nonspecific, radiological findings, may hinder the suspicious of MBD and highlights the importance of considering alternative diagnosis. Wernicke encephalopathy usually presents with involvement of the medial thalamic nuclei, hypothalamus, mamillary bodies, and periaqueductal grey matter. Pontine and extrapontine myelinolysis can also be differentiated by the involvement of the central pons, basal ganglia, thalami, lateral geniculate body, cerebellum, and cerebral cortex. Ischemic stroke, contusion, multiple sclerosis, lymphoma, and other diseases with preference for the deep white matters and corpus callosum may mimic MBD syndrome, but usually can be differentiated by the asymmetrical appearance of their lesions, once symmetry is an key feature for this condition^7,8^.

The cornerstone of treatment is supplementation of complex B vitamins, especially thiamine, as well as cessation of alcohol consumption along with rehabilitation therapy. The use of high-dose corticosteroids in acute patients has been reported as a safe strategy with positive outcomes^2,5,9,10^.

In the clinical case described, the patient was hospitalized and started on intravenous thiamine replacement, medications for alcohol withdrawal syndrome, as well as treatments to manage symptoms of depression/anxiety. In subsequent follow-up visits, the decision was made to refer him for joint follow-up with outpatient psychiatric care, while continuing surveillance with periodic exams and vitamin replacement, despite only slight improvement in neurological symptoms.
